# Globalisation and transitions in abortion care in Ghana

**DOI:** 10.1186/s12913-019-4010-8

**Published:** 2019-03-21

**Authors:** Patience Aniteye, Susannah H. Mayhew

**Affiliations:** 1 School of Nursing, College of Health Sciences, University of Ghana, Legon, Ghana; 2 Department of Global Health and Development, London School of Hygiene & Tropical Medicine, 15-17 Tavistock Place, London, UK

**Keywords:** Abortion, Ghana, Globalisation, Policy, Policy implementation

## Abstract

**Background:**

Access to safe abortion is a globally contested policy and social justice issue – contested because of its religious and moral dimensions regarding the right to life and personhood of a foetus vs. the rights of women to make decisions about their own bodies. Many nations have agreed to address the health consequences of unsafe abortion, though stopped short of committing to providing comprehensive services. Ghana has a relatively liberal abortion law dating from 1985 and has ratified most international agreements on provision of care. Policy implementation has been very slow, but modest efforts are now being made to reduce maternal mortality caused by unsafe abortions. Understanding whether globalisation has played a role in this transition to practice is important to institutionalise the transition in Ghana and to learn lessons for other countries seeking to implement policies, but analysis is lacking.

**Methods:**

Drawing on 58 in-depth key informant interviews and policy document analysis we describe the development of *de jure* law and policies on comprehensive abortion care in Ghana, de facto interpretation and implementation of those policies, and assess what role globalization played in the transition in abortion care in Ghana.

**Results:**

We found that an accumulation of global influences has converged to start a transition in the culture of abortion care and service provision in Ghana, from a restrictive interpretation of the law to facilitating more widespread access to legal, safe abortion services through development of policies and guidelines and a slow change in attitudes and practices of health providers. These global influences can be categorised as: a global governance architecture of reproductive rights-obligations which creates pressure on signatory governments to act; and global communication of ideas and mobility of health providers (particularly through cross-cultural training opportunities and interaction with international NGOs) which facilitate global cultural interaction on the benefits of safe abortion services for reducing consequences of unsafe abortions.

**Conclusion:**

Globalisation of information, debate and training experience as well as of international rights frameworks can together create a powerful force for good to protect women and their children from the needless pain and death resulting from unsafe abortions.

## Background

Abortion is a global phenomenon carried out for diverse reasons and with an array of consequences that affect women, their families, societies and health systems [1]. By World Health Organization (WHO) standards, abortions procured in locations with minimal medical standards and/or provided by unskilled health professions are defined as *unsafe* [2, 3]. The global burden of unsafe abortion is huge with 49% of all abortions being unsafe; in Africa more than 97% are unsafe [4]. Unsafe abortions may result in outcomes with enormous cost to health services [5]. Globally 13% of all maternal deaths are attributed to unsafe abortion and almost all abortion-related deaths occur in developing countries with the highest number in Africa [4]. Many more women who survive experience morbidity and disability that diminish their quality of life [3].

In Ghana unsafe abortion contributes to the high maternal mortality rate of between 310 and 402 deaths per 100,000 live births [6]. Yet unsafe abortions are preventable and the solutions, including sex education and effective family planning to avert unplanned pregnancies, are well documented, practical and affordable [7]. More directly, Comprehensive Abortion Care (CAC) which provides access to safe abortion and post-abortion care, is strongly advocated by the World Health Organisation as best practice [3]. Even donors who will not support safe abortion services (like USAID) do support post-abortion care as part of a comprehensive approach [8]. The situation in Ghana is changing slowly. In 1997 a study in four regions in Southern Ghana estimated there were 17 induced abortions for every 1000 women of childbearing age [9]. In 2007 the nationally representative Ghana Maternal Health Survey found that 15% women aged 15–49 had ever had an induced abortion with 5% having one in the five years preceding the survey [10]. In 2017 the survey found 20% women 15–49 had ever had an abortion, with 7% reported one in the preceding five years [6]. This small increase may indicate increasing awareness of services.

Globalisation refers to interconnectedness and interdependence of the human community. Events in one nation increasingly appear to have ripple effects (negative or positive) on other nations globally [11], both at a national, policy level and the individual, social level. Access to safe abortion is a globally contested policy and social justice issue – contested because of its religious and moral dimensions vis-à-vis the right to life and personhood of a foetus vs. the rights of women to make decisions about their own bodies. International consensus meetings have been held to define and agree action on unsafe abortion. At the International Conference on Population and Development (ICPD) in 1994, governments were urged to ensure widespread availability of post abortion care (PAC) and safe abortion services; the latter to be delivered within the framework of national abortion laws. Many nations agreed to address the health *consequences* of unsafe abortion, though stopped short of committing to providing comprehensive safe abortion services [1]. The Fourth World Conference on Women in Beijing in 1995 called on governments to review restrictive abortion laws in line with protecting the rights of women. Consequently, between 1995 and 2003, many countries in Africa where most abortion laws are restrictive, including Benin, Burkina Faso, Chad, Guinea and Mali, reformed their laws to allow for abortions where pregnancies result from rape, incest, foetal impairment or risks to women’s health [12].

Legal reform is important because there is a strong correlation between restrictive abortion laws, high levels of unsafe abortions and high maternal mortality and morbidity [1]. On the other hand, in countries, where laws and policies allow abortion under broad indications, the incidence of, and mortality from, unsafe abortions are relatively low. Worldwide, abortion is permitted in law for social or economic reasons in only 16% of low income countries as opposed to about 80% of high income countries [3]. There are many influences on abortion laws: depending on the country these may include civil, common and religious laws, which in many African countries including Ghana, frequently derive from the inherited laws of their colonisers [13].

In Ghana abortion was restricted under common law, being criminalised in the penal code, as inherited from the UK colonial government. During the years of military dictatorship in the 1980s, however, when the influence of institutionalised religion was perhaps lessened, this law was modified and liberalised. The 1985 law (Government of Ghana PNDC Law 102) permits abortion in cases of rape, incest, foetal abnormalities or where the pregnancy is a risk to the woman’s physical or mental health, although these remain exceptions within in the criminal code. Since then, Ghana ratified most of the international treaties and has made modest efforts at reducing maternal mortality by tackling the problem of unsafe abortions. Post abortion care forms an integral part of the Safe motherhood initiative that was implemented in 1990, though operational service guidelines were only finalised in 2006. The reality of the provision of accessible safe abortion services has been very different. Historically safe abortion services were largely available in private clinics so not financially accessible to all women. Some abortions were provided clandestinely in public hospitals and were hard to access. Over the past decade, however, changes have occurred at the level of individual providers, in line with the law, and safe abortion services are now being provided more frequently in health centres and public hospitals by obstetricians and midwives who are trained and willing to provide the services, though coverage remains patchy [14]. Understanding whether globalisation has played a role in this transition to practice is important to institutionalise the transition in Ghana as well as to learn lessons for other countries seeking to implement policies, but analysis of globalisation elements in this respect is lacking. The purpose of this paper is to draw on key informant interviews and document analysis to describe the development of *de jure* law and policies on comprehensive abortion care in Ghana, the de facto interpretation and implementation of those policies, and assess what role globalization played in the transition in abortion care in Ghana.

## Methods

To gather rich data we utilised an in-depth qualitative design that included detailed interviews and also a policy document review. Ethical approval was obtained from the London School of Hygiene & Tropical Medicine, where the study was designed, and from the Ghana Health Service. Data were collected only after obtaining written informed consent from each respondent. Because of the nature of the topic, and the stigma attached to it in Ghana [15], protecting the confidentiality of participants was a primary consideration.

Fifty-eight in-depth interviews were conducted between November 2006–July 2007. Respondents had both knowledge of and interest in the issue of abortion care and included obstetricians (*n* = 15), midwives (*n* = 14), other health professionals (pharmacists and trainers) (*n* = 12), policy makers (parliamentarians and MoH officials) (*n* = 14) and three (3) representatives of development agencies/NGOs.

Health professionals were purposively sampled from a range of public and private facilities in the Greater Accra Region, from MOH list of facilities in the region. All the health centres had units (reproductive and child health (RCH) and family planning (FP)) that offer reproductive health services where women with abortion complications are treated. Staff were identified with the help of the unit/facility heads and were selected because they had substantial knowledge, exposure and experience of abortion. In addition to obstetrician/gynaecologists, who provided clinical abortion services, midwives (rather than nurses) were included because it is they who staff the RCH units providing antenatal, post-natal and family planning services where women in need of reproductive health care services most commonly present. Pharmacists were included in the study because in Ghana studies have shown that community pharmacy shops sell abortifacients (e.g., Cytotec or Misoprostol) and are the first point of call when women have an unwanted pregnancy since abortion services are not openly available in public hospitals and private clinics are very expensive. Health professionals involved in training were also included. Policy makers included parliamentarians (7) and MoH officials (7) and were purposively selected based on their involvement in or knowledge of abortion policies and services.

Interview questions and prompts were based on the background of each participant but all were asked about their knowledge of the abortion law and how they regarded unsafe abortion. Interviewees were probed on their sources of knowledge and the reasons for the views and attitudes they held. This included discussion of training and information from other countries, knowledge and perceptions of international treaties and abortion-related laws in other countries. Further details on the research instruments have been published elsewhere [15].

Each semi-structured interview lasted between 60 and 90 min. All interviews were conducted in English and all but one audio recorded and transcribed verbatim. The one that was not recorded was fully transcribed from notes immediately after the interview. Field notes captured all that transpired during the interview including the body language of the participants. Data were analysed using content analysis with the assistance of the qualitative software NVIVO Version 6 (QSR International) and Framework Analysis was used for analysis after code clusters from the software had been exported from the software into excel sheets for manual analysis. One researcher (PA) analysed all interviews in depth in consultation with two other researchers (in particular SM). Transcripts were repeatedly read and recurring themes noted and grouped. Themes were recorded and scrutinized for patterns. Based on identified patterns, the themes were grouped in a hierarchical manner. A code frame was developed and used to index the entire data set. Following indexing, all data under a sub-theme were pulled together and descriptive accounts were written on each sub-theme.

Documents critically reviewed included the abortion law as well as the policy documents of the Ministry of Health and the Ghana Health Service on reproductive health and related to the topic under investigation. The key policy documents included ‘*The Criminal Code of Ghana’ (GoG, 1985 Amendment), ‘The* N*ational Reproductive Health Service Policy and Standards’ (GHS, 1996 and 2003)*, ‘*The Prevention and Management of Unsafe Abortion: Comprehensive Abortion Care Services Standards and Protocols’ (GHS, 2006)*.

Credibility or trustworthiness, an important hallmark of qualitative inquiry, was ensured through: respondent validation, where transcripts of respondents were shown to them to ascertain whether what they said have been correctly represented in the transcripts; a conscious search for and analysis of deviant cases; and an audit trail, which refers to a record of all decisions made to guide data collection and analysis as well as a record of researchers’ biases and prejudices about the study topic before, during and after data collection. Trustworthiness was also supported through triangulation of data sources and methods. Data sources (health providers; policymakers; other key informants) and data collection methods (in-depth interviews and document analysis) were used to confirm and ensure completeness of the findings. The researcher’s prolonged field engagement (nine months, in addition to personal knowledge of the context) and checking the correctness of findings with participants supported credibility.

## Results

### The abortion law of Ghana and its interpretation

The abortion law in Ghana does not stand-alone but is within the Criminal Code (Act 29; 1960). The abortion law code was amended in 1985 (P.N.D.C. Law 102). Abortion is legal when pregnancy is thought to occur due to rape or incest and where pregnancy continuation is deemed detrimental to maternal life or injurious to her physical or mental health. Abortion is also legal when the foetus has an abnormality, but abnormalities are not specified. This law was enacted during a military era so the actual process was not made public. One policy maker said:

*“ … when this law (PNDC Law 102) was passed by the military, whether it was because they were fearing the opposition of the Catholic Church I don’t know; but they passed it and they didn’t make any noise about it at all so it took some of us a while before we realized that this thing had been done.”* ***Policy Maker 1***

Because the process of legal development lacked transparency, knowledge of abortion law (even 20 years after it was enacted) was not well disseminated and varied considerably among respondents. The policy makers, lawyers and obstetrician/gynaecologists, who were usually the more highly educated respondents, were more knowledgeable than were the less-highly educated nurse-midwives and most of the religious leaders who were often dogmatic on the issue. In general, respondents’ attitude to and interpretation of the abortion law depended largely on their stance toward abortion (as distinct from abortion services). Religion and social norms about morality clearly played a role in influencing their attitudes towards safe-abortion services but it became clear that global influences had also played a role.

Those who interpreted the law liberally and were supportive of it tended to be better educated and more aware of international agreements and debates on reproductive rights. Almost all the medical doctors who supported safe-abortion service provision had been at least partially educated abroad, mainly in the former Soviet Union or in Europe, where abortion access is highly liberalised. Many cited international agreements on human and women’s rights in relation to this as well as referred to their practices overseas where safe-abortion services were seen as the norm.

*“When you look at the Cairo Conference, it was stated categorically that it is the right of the woman to decide … Also, it said where abortion is not against the law, it is the right of the woman to access safe-abortion services so it is a human right … those of us in obstetrics put the right of the woman above that of the foetus … ”* ***Obstetrician 13***

*“ … in Eastern countries and Europe, I know they have such facilities. Even single women who get pregnant from casual relationships can go for the service because over there they are so careful about their population and individuals choose to have children when they want. But here, when one gets pregnant, we have a simple understanding that it is God who has given the child. So even if you are not ready you should have the child ... on moral ground, we encourage most women who get unplanned pregnancies to have the children”.* ***Other Health Professional 5***

These respondents unequivocally described abortion in Ghana as legal for the cases prescribed by the amended law. They emphasised Section 58; Sub-section 2, which highlights the legal exemptions rather than the criminal nature of the procedure. The mental health clause was singled out by some participants as one that allows abortion within a very wide scope:

*… very liberal … you can do a lot within the confines of the law. Those of us who are in favour of provision of safe abortion because of the problems of unsafe abortion we have seen, think we can use the mental health of the woman; like, the woman comes to say, if I carry on this pregnancy, I will commit suicide … so we can capitalize on those aspects … physical or mental health of the woman … it is liberal. The only thing is that people don’t know the law.* ***Obstetrician 4***

Some of those objecting to abortion services also highlighted the liberality of it – but as a negative thing:

*“ … the law we have in this country is too wide. [ … ] The worst part of our law is the period of gestation. There is no limit. As far as the woman has not delivered, you can terminate the pregnancy* ***.” Obstetrician 15***

While others considered the abortion law’s inclusion in the criminal code as meaning it is restrictive and must not be provided:

*“ … It is the fact that the abortion law has been put in the Criminal Code … A lawyer said the fact that they have put it in a Criminal Code means it is a crime unless it is done under some provisions... a lawyer gave that interpretation”.* ***Obstetrician 13***

Those who were opposed to provision of abortion services perceived them as illegal under the law, usually citing Section 58, Sub-section 1, which describes abortion as a criminal offence. They focused on the 1960 pre-amendment version of the law which stated that only exemption was risk to the mothers’ life. This, in their view, was the only reason for which abortion could be permitted; it served as a last resort. A few emphasized that even in cases of rape, incest or foetal abnormality, a pregnant woman could be required to deliver her baby. Almost all those holding this narrow interpretation were midwives with limited exposure to international debates or alternative views or training on safe abortion – their point of reference was almost always their religion. The following quote was typical:

*“ … I am against abortion because God says keep your bodies as a holy temple for me to come and dwell in you … I wouldn’t want to offend my God. [ … ] When you do it [abortion], your hands become bloody.”* ***Midwife 8***

Other participants pointed to the ambiguities that continue in the Law, describing it as having loopholes and grey areas. Gestational age limits, proof of rape, the nature of maternal risk, what constitutes physical and mental health and referral processes were all mentioned as important issues that are not included or are not clear within the law.

### Development of the policy and influences on national implementation guidelines

#### Policy content

The *National Reproductive Health Service Policy and Standards* was intended to offer direction to Ghanaian organizations providing reproductive health care [16]. The policy covered strategies for safe motherhood in detail. The strategies outlined included provision of essential obstetric care, but unsafe abortion was not discussed.

A policy review was conducted and the Standards updated in 2003 to address these important omissions and to incorporate changes in care [17]. Part 1 covers the Reproductive Health Service policy. The sections relevant to comprehensive abortion care included the nine service components of reproductive health care including prevention and management of unsafe abortion and post abortion care. Emergency contraception and not using abortion as family planning strategy were two important considerations emphasized in the document. ‘Prevention and management of unsafe abortion and post abortion care (PAC)’ included all of the following; family planning counseling and services, abortion services in accordance with the law, managing and referring abortion complications, linking post abortion care to other related healthcare services, creating awareness of dangers of unsafe abortion and educating women regarding complications of abortion. Educating the *public* regarding obtaining safe, legal abortion was not included.

Detailed reproductive health standards were included. Under prevention and management of unsafe abortion and post abortion care, history taking, resuscitation, referral, manual vacuum aspiration provision to family planning and counseling were listed as strategies skilled providers employ to manage and follow up women who had experienced unsafe abortion. The comprehensive abortion care standards and protocols [18] permits midwives and physicians to provide manual vacuum aspiration at all levels of institutional care.

#### Influences on policy development

The Policy review instigated in 2003 may have originated from global endorsement of the need for safe abortion [12] and from activities of global institutions in Ghana. A WHO call on governments and health systems to provide safe abortion services where permitted by law and the provision of technical and policy guidelines by the organization for health systems came out three years before [19]**.** Moreover, an increasing number of international NGOs committed to safe abortion (including Marie Stopes International and Ipas) became active in Ghana during the 2000s. Some respondents cited the involvement of Ipas in particular as a critical motivation for the policy review regarding safe abortion services which was triggered by Ipas’ organisation of a conference for African Ministers of Health in Addis Ababa, Ethiopia in 2003 and attended by a Ghanaian delegation. Ipas is “a global nongovernmental organization dedicated to ending preventable deaths and disabilities from unsafe abortion”.

In March 2003, Ipas organized a meeting in Addis Ababa, Ethiopia to take *‘Action to reduce maternal mortality in Africa’*. The WHO document entitled *‘Safe abortion: technical and policy guidance for health systems’* [17] was discussed at length and its usefulness and timeliness were highlighted. This document described how countries could ensure access to safe abortion services to the extent permitted by law. As part of their commitment to help reduce preventable deaths of women in their fertile age, a communiqué was signed by all the attending countries to take action to reduce maternal mortality, using the WHO prescribed document. Taking inspiration from the deliberations of the meeting in Ethiopia, Ghana formed an interdisciplinary reproductive health group to implement the meeting’s recommendations. A task force was thus formed at the Ghana Health Service following the Ipas meeting.

The Ghana Health Service taskforce was asked to develop a strategic plan to address the problem of unsafe abortion in Ghana. In December 2003, the plan was adopted. The GHS, with significant technical support from Ipas and MSI, revised the National Reproductive Health Service Policy and Standards in the light of this plan, incorporating an additional objective under: *‘Prevention of Unsafe Abortion and Post Abortion Care’* allowing for provision of safe abortion services to the full extent of the law. The WHO 2003 guidelines and the Ipas 2003 meeting served as an impetus for the 2003 Policy and Standards revision. The document provided, for the first time, clear descriptions of CAC and PAC provision at different service delivery levels. In May/June 2005, a strategic assessment of abortion and abortion care services was conducted led by GHS and an advisory committee of policy-makers [20]. The purpose of the assessment was to enable key stakeholders to discuss issues of interest in reproductive health including abortion. The assessment, commissioned by the Ministry of Health, was conducted by a 17-member team some of whom were obstetricians and midwives and some were representatives from Ipas and other international NGOs. Major findings included poor knowledge of the abortion law among the public and many health providers and the dangerous methods used by women and girls to induce abortion. Other findings were the strong *‘culture of silence’* surrounding abortion and the current willingness of communities to openly discuss abortion issues. High costs of abortion services and the clandestine nature in which services are provided were identified as well as the increase of medical abortion in urban areas. These findings from the 2005 strategic assessment supported the findings on the abortion law that emerged from the interviews of service providers [21].

Based on response to the expanded objectives in the GHS 2003 Reproductive Health policy to provide abortion services to the extent permitted by law, and to help address the findings of the 2005 strategic assessment, *the Standards and Protocols for the Prevention and Management of Unsafe Abortion* were developed in 2006 [16], again with support from IPAS and MSI. A critical “window of opportunity” had opened when the leadership of the Ministry of Health’s Reproductive Health Unit changed and the incoming head was much more sympathetic to the need for safe abortion services to tackle maternal mortality. Two respondents, an obstetrician- gynaecologist and a lawyer-parliamentarian, were themselves involved in both the policy and guidelines development and its amendments and were active in insisting the inclusion of a clause allowing the provision of safe, legal abortion services in the new Ghanaian document. One highlighted the change that has taken place in recent years:

*“ … When we were writing the [original] policy, the issue of termination of pregnancy was … actually rejected!... when people were talking about safe motherhood, it was not one of those areas they wanted to talk about until it became a thing that it’s a big topic which accounts for nearly a quarter of your deaths so if you don’t do anything, you can’t reduce your maternal deaths so everybody is beginning to realize that it’s a subject … the latest review has included termination of pregnancy within the limits of the law. A protocol has recently been developed”.* ***Obstetrician 7***

INGOs had a significant input too. Their already close collaboration with GHS on strengthening service-delivery put them in a good position to capitalise on the more sympathetic stance within the MoH leadership and help the GHS develop the 2006 guidelines to provide technical and managerial guidance for the provision of comprehensive abortion care services of desired quality. This was further revised in 2012. Policy makers and other respondents involved in the development of the guidelines highlighted this critical involvement from global organisations:

*“ … We had support from Ipas. WHO supports because they came out with a document addressing unsafe abortion with the methods and ways it should be done … it came from ICPD [International Conference on Population and Development] that “where abortion is not against the law, provide the service” … That is where the thing started that we have to address … they [Ipas Ethiopia meeting delegates] came back and we were asked to come up with the strategic plan to address it [the problem of unsafe abortion] … ”* ***policy maker 2***

As well as the influence of global institutions and documents on the trajectory of abortion policy in Ghana, the exposure of influential national stakeholders to practices and debates in other countries and to the content of international treaties and declarations, had an influence on their own perceptions of the Ghanaian situation. As noted in the discussion of the Law, many participants who trained outside Ghana expressed the need for abortion care, based on what they had seen in other countries. One parliamentarian, recognising global influences on social norms, said Ghana needed to follow the example of other countries if it is to make any headway in saving women’s lives from unsafe abortions:

*“ … Society is dynamic. This nation has come of age; we are celebrating 50 years the whole world has been reduced to a global village, communication … We need to be in tune … We need to move with time irrespective of our beliefs, religious and other things … we are in scientific world and there is the need for the nation to have a second look at that matter regarding abortion, whether it should be legalized or not … ”* ***Policy Maker 9***

Some respondents attributed the transition to a more accessible comprehensive abortion packages in Ghana to the movement by the self-proclaimed “reproductive health group” a group of stake-holders, led by Ipas, who worked quietly to train, equip and ensure that there was a critical mass of health providers competent and willing to provide safe, legal abortion services in public health facilities. Others noted the initiatives of the Ministry of Health and Ghana Health Service in response to international obligations to ensure the safety of women procuring abortions. A number of respondents also spoke of Ghana’s obligations under international treaties including basic human rights:

*“ … There’re too many women dying from unsafe abortion in our hospitals. We have signed on to the MDGs; we have to reduce maternal mortality”* ***Obstetrician 6***

*“I raised some concerns with respect to abortion … our laws allow abortion in certain circumstances so you can’t have a policy, which is inconsistent with the law. When you have that you’re violating human rights of people (women).”* ***Policy Maker 9***

Another obstetrician highlighted his position regarding the rights of women:

*“When you look at the Cairo Conference, (ICPD), it was stated that it is the right of the woman to decide … Also, it said where abortion is not against the law, it is the right of the woman to access safe abortion service so; it is a human right … ...”* ***Obstetrician 13***

The holistic WHO definition of health was often used by respondents as a basis for providing services for women with unwanted pregnancy:

*“ … Once it [the law] mentions physical and mental health, the grounds are wide. Because the WHO definition of health is a very wide definition; not only physical incapacity but mental and general wellbeing... it is for the doctor to believe that woman and decide … ”* ***Obstetrician 1***

#### Implementation of the policy

Post-abortion care (PAC) has been visible in Ghana for much longer than comprehensive abortion care (CAC). The MOH and PRIME^1^ integrated PAC into Safe Motherhood clinical skills training in three Ghanaian regions in 1997. PAC was included in the National Reproductive Health Policy and Standards under *‘Prevention of Unsafe Abortion and Post Abortion Care’*. Pre-service post abortion care training is part of the medical curriculum although pre-service training for nurse/midwives includes only post abortion Family Planning. In an assessment of the readiness of selected facilities in three regions in Ghana in 2005–6 to offer contraceptives and comprehensive abortion care, the availability of post abortion care and CAC was described as low in the health centres and polyclinics in three regions (Aboagye et al. 2007). Of 74 polyclinics and health centres, 7(12%) offered PAC and only 1(1.3%) CAC. With respect to hospitals, 15 (93.7%) of 16 offered PAC and 11(68.7%) CAC. None of the regions satisfied the minimum requirement of five basic sites per 500,000 population [22]. It is evident that both PAC and CAC services are not widely available with CAC appearing to be much less available than PAC.

Among our respondents, the vast majority of policy-makers, who were doctors based at the MOH/GHS headquarters, and most of the practising obstetrician-gynaecologists interviewed, had some knowledge about the reproductive health policy and practice guidelines. Two admitted that even though they had been practising obstetrics for close to a decade, they only read these thoroughly ahead of their (recent) post graduate examinations. Two groups of health workers who had very little or no knowledge about the policy or its guidelines were the nurse-midwives and pharmacists. There were some midwives who knew nothing about the policy. Clearly most providers *‘on the ground’* had very little knowledge. Those who knew the policy were either directly involved in its formulation and review or had heard about it at workshops.

INGOs are, again, a significant influence on policy implementation. In 2006 (the year the *Guidelines* were finally published) the five key INGOs working on safe-abortion came together to form the R3M programme to support expanded access to safe abortion and skilled treatment of complications from abortion. Under R3M each INGO coordinated to take on a specific role to avoid duplication and maximise impact on improving provision of abortion and post-abortion care services – activities which continue today. Two worked to support abortion and post abortion care in the public system through advocacy/policy engagement and infrastructure/training support. Another worked through its own clinics and its system of franchisees to provide abortion and post-abortion care in the private-for-profit sector. A fourth focused on demand creation through community awareness raising, case follow up and social marketing. A fifth provided coordination, training, monitoring and evaluation. One provider summed up what many others also said – that without INGOs training for implementation would not have happened:

*When you have a policy, you need the standards and protocols for that policy to be implemented, but we have not had it, until … it was 2006 or so … but there were no standards and protocols. The policy was just there; paper work. It was just paper work. Nobody was implementing it, until [INGO’s name] championed the course and eventually they have been able to help the government.* ***Obstetrician 13.***

Another noted:

*I think this comes about as a cooperation between international NGOs and those employed to be in the reproductive health department of the MOH [ … ] I think there is a heavy guidance by international organizations which are well meaning and want to provide funds for certain things (safe, legal abortions and related services) to be done.* ***Obstetrician 1***

One of the policy makers who was also involved in training noted the importance of “values clarification” exercises, introduced by INGOs, for allowing providers to be exposed to different arguments about abortion-care and help them make up their minds:

*“We’ll have a lot of values clarification exercise and then we’re given scenarios … we have the strongly agree, disagree in between so people stand in different places. And they’re given a topic or statement, something like “abortion services should be provided in the facilities”. And we have strongly agree, on the fence, disagree and strongly disagree. And then you all stand in your places. And then you argue the case out and then you’ll find out that some people will move from the “disagree” to the “agree” or some people will move from standing on the fence, they’ll take a stand. They’ve been able to get information and internalize. It’s not a one-day thing but at least that one-day workshop does a lot. We met at the Nurses and Midwives Council and had it with them. We also had it with some of our stakeholders. Ipas spearheaded this. We have a lot of technical assistance from Ipas on values clarification. But we have people in the country who have also been trained on values clarification so they‘re part of the team of trainers. So in all these facilities we have values clarification exercises. It will not be just the people who will provide the service; even the gate man, I mean people will have to be orientated. [ … ] But you also have the right to conscientious objection [ … ] but then you’ll not block someone from getting a service that could save their lives”.* ***Policy maker 2***

The other key influence to emerge was exposure of health providers to abortion-care provision in other settings. Many obstetrician-gynaecologists have worked, trained or travelled abroad professionally and their attitudes were striking:

*“I worked in the UK for several years and I offered terminations and you are not paid for it, but it is just a service you are providing and because you believe in it that if you don’t do that maybe something worse will happen. We all know it from the history of abortion law in the West as to how much drastically this reduces maternal mortality and maiming of people from back street abortions so if you want to take it in that direction and you morally look at it as, yes, this is service I want to provide, it as part of my specialty …*.” ***Obstetrician 1.***

*“Of course, in our Africa societies, again it is the same teaching that it is a sin. They also have the same respect for life. But western science or those countries where abortion is liberal, we cannot say they don’t have respect for life. All these people have respect for life but it depends upon how you interpret it. The way I go around it is that, we always look at the greater good; we have to be seen to be doing the greater good”.* ***Obstetrician 13.***

These findings suggest that globalisation, or exposure to its processes, has influenced both the development and de facto implementation of policy guidelines on safe abortion in Ghana. The patchy availability of abortion services seemed very much to depend on the exposure of individual providers to INGO-sponsored training and/or to other global debates and globalisation processes affecting social norms on abortion and abortion services.

## Discussion

We have described the development and interpretation of the law and policies addressing access to abortion services in Ghana, with a view to illustrating the influences on these and the role that globalisation has played in shaping the transition from restrictive access to abortion services to more equitable access.

Ghana’s legal framework on abortion, the 1985 amendment which was the result of a dictatorship, was in a way ahead of its time (it is supported by the Cairo Platform of Action nearly a decade later) – despite being in the criminal code (which avoids more controversial discussion of “legalisation”) it is vague and liberal enough to allow practitioners to work within it (again without attracting too much attention). This can have negative consequences too whereby legitimate providers are not aware of the wide circumstances within which they can legally provide abortion services. Furthermore, the social and political climate in Ghana until around 2006 was such that the law was never operationalized into policy guidelines. The fact that guidelines were eventually developed is partly due to a range of socio-political global influences as Ghana consolidated itself as a multi-party democracy with vibrant economic growth and global social exchange.

A number of frameworks have emerged for analysing the globalisation of influences on health, though none particularly focused on the policy level. We found a useful one to be that suggested by Huynen et al. (2005) in *Globalisation and Health* which was developed to categorise the global influences on population health [23]. Four of the six hypothesised “features” are relevant to our policy case study on abortion (global environmental influences and global markets are not relevant).

First, *new global governance structures*, defined in the Huynen paper as globalisation influencing the interdependence among nations as well as the nation state’s sovereignty which leads to new global governance structures. In our case study “new” governance structures have developed in relation to the right to health. International treaties and declarations, including those arising from the 1994 International Conference on Population and Development (ICPD) and N’s 1995 Beijing Conference on Women, embed a shifting understanding of legal rights that protect reproductive choices for women. Ghana’s signing of these international agreements holds weight beyond sovereign legislation, creating an internationally witnessed obligation of the signatory government to be seen to respond in its own laws and policies. In fact Ghana’s laws predated its signing of the international agreements but the signing added weight to the need to translate legal rights into practical policies and guidelines – a process that took another 10 years and came about because of pressure – and action – from international NGOs as well as a variety of other global exchanges. The re-imposition of the Mexico City Policy – or Global Gag Rule –by the present US administration post-dates our fieldwork, has made the environment more difficult for related service providers. The “gag” rule refuses funding to NGOs that provide information about or services for safe abortion. Although Ghana did not suffer unduly from the previous gag rule imposed by President Bush Junior it did result in the Planned Parenthood Association of Ghana, which received significant USAID funding, having to withdraw outreach services and lay off workers as a result of losing funding through not signing the “gag” rule. However, since safe abortion services in Ghana are largely provided by government clinics and pharmacies we would not expect it to have a significant effect on availability of safe abortion services, though users of NGO-provided services may well find their choices more limited.

Second, *global communication*, defined as globalisation making the sharing of information and the exchange of experiences around common problems possible. In our case study some respondents had been exposed through training and their own use of the internet, to more liberal views of abortion which they claimed had changed the way they viewed safe abortion service provision. The communication of global scientific evidence and debate persuaded them that saving women’s lives (as opposed to foetuses) was an important, though a difficult choice. The woman is the full person alive now who might die without access to safe abortion and then not only she but her other existing children would suffer, thus the greater good would be to allow women a choice. This has helped to temper the negative influences of institutionalised religion. We have shown elsewhere that while doctrinal religious beliefs contribute to stigmatisation of abortion providers, this is mainly feared by lower-educated practitioners who have had little training on or exposure to more liberal social and medical norms in other countries [15].

International NGOs have been critical players in Ghana for communicating – and advocating on – these global rights perspectives and frameworks. For example, R3M, taking up global debates on abortion rights pushed for evidence, reviews and piloting of interventions as well as providing training and support to influence service provision in Ghana. Ipas was particularly influential at a global level, having a big role coordinating the Addis Ababa meeting for all African Ministers for Health and in supporting the national follow-up after those agreements – to which Ghana was a signatory. WHO added its weight as a global agency tasked with disseminating best practice and service-delivery guidance.

While global communication has clearly contributed to a transition in care practices among Ghanaian providers, the same cannot be said for women and girls who should be the beneficiaries of that care. In 2017 the Ghana Maternal Health Survey found that only 11% women knew that abortion was legally available in Ghana [6]. This may explain the apparent lack of significant change in women accessing clinic-based services. In 2017 38% of women undergoing abortion reported using medical pills; 34% used a clinic-based medical procedure (mostly dilation and curettage or dilation and evacuation) but 27% still used non-medical methods [10]. This appears to be only a fractional decrease from the findings from the (non-representative) 1997 study from Southern Ghana in which 32% respondents used non-medical methods, the rest using clinic based staff or pharmacies [9]. Nevertheless, it should be noted that respondents in southern Ghana will have had significantly better access to services than their counterparts in the underserved middle and northern regions of the country, so a nationally representative percentage would have been lower. Furthermore, in the past, the clandestine provision of “safe” services by clinicians but for private fees was widespread which may account for a large part of the “medical” services reported in the earlier study. What this shows is a clear need for communications to focus on improving women and girls understanding of their rights to safe, legal abortion services that are increasingly available in Ghana.

Third, *global mobility*, defined as a major increase in the intensity and velocity of movement and by a wide variety in ‘types’ of mobility. In our case study the higher trained specialised cadres of staff (obstetrician-gynaecologists) had been trained in Europe (mainly Eastern Europe) where they had been exposed to, taught about, and practiced provision of, safe abortion services. Various American schools have also established advanced training programmes, including the University of Michigan Department of Obstetrics and Gynecology began postgraduate training of Ghanaian obstetricians in 1986. One graduate is quoted as saying: “*You saw how things are being done in the developed world. It gave me an opportunity to compare methods there with those here. And I concluded that after all, what they are doing in the developed world is not miles ahead of what they are doing here, so I became more confident in what we are doing as OB-GYNs.”* [24]*.* Like most, if not all, international training programmes, these opportunities are not available to lower cadres of staff. In our study it was noticeable that the midwives, lower in the medical hierarchy, who had not had such opportunities consequently had much more limited exposure to different approaches, debates and practices. Their views were correspondingly more traditional and more defined by doctrinal religious beliefs. Nevertheless, there are also attempts in-country to build capacity more broadly for reproductive health care provision. For example, the University of Michigan in collaboration with Ghana College of Physicians and Surgeons and two teaching hospitals established International Family Planning Fellowship Program in 2008, to locally train fellows in family planning and reproductive health.

Finally, *cross-culture interaction*, defined as the globalisation of cultural flows resulting in interactions between global and local cultural elements. This is linked to the previous two categories – communication and mobility facilitate the interaction of different cultural perspectives. In relation to reproductive rights this means that international norms of acceptability of these rights become normalised in local settings through the sharing of ideas and the gradual change of perspective, particularly when that is combined with experience of the health benefits of a different approach. International NGOs, neglected in the original theory, are an important conductor for intercultural interaction. Most are headquartered in developed countries but maintain country offices. Their international staff bring with them globally-informed ideas and practices. In our case study the INGOs active in the abortion field were providing services, supporting training to public sector staff and advocating to government agencies to bring their policy guidelines in line with both their own legal position and their obligations under international declarations. In Ghana the country offices of the international NGOs are usually staffed by Ghanaians making them appear more “home grown” but advocating strongly on the lines of internationally agreed obligations. Another complementary study shows how the INGOs working in Ghana on sensitive abortion issues took a “softly, softly” approach, getting the ear of key policy and social thought-leaders, focusing on preventing the consequences of unsafe abortion rather than pushing for controversial “rights” [25].

In Ghana the law itself was not sufficient to achieve a change in practice, partly because its embedding in the penal code makes it appear inaccessible, but an accumulation of global influences has converged to start a transition in the culture of abortion care and service provision among those cadres of health providers who have interacted with other cultures. The existence of policy implementation guidelines, whose formulation was led by INGOs, means that there is now, additionally, a clear professional obligation on all cadres of staff to transition to a more equitable, non-judgemental provision of abortion services.

There is a debate in the globalisation and health literature on the extent to which globalisation is helpful or harmful to health. Richard Feachem in his 2001 British Medical Journal article bucked the trend when he suggested it was mostly helpful [26]. This was hotly contested by others who pointed to neo-colonialist tendencies exacerbated by globalisation ([27, 28] and many others in the Letters section of British Medical Journal Vol. 324, 5 January 2002). Some years later Schrecker et al. in the Lancet (2008) held that globalisation creates inequities for health (as well as more generally) [29]. Our case study provides very clear refutation of this in relation to abortion. We show that where cultural interactions on health and rights issues, global mobility of staff and global governance architecture converge to protect contested rights for vulnerable people, then globalisation can very clearly work in favour of these rights and create more equitable access to stigmatised or hard-to-access services, particularly for the poor. This view is supported by London and Schneider (2012) who argue that global human rights provide a normative framework enabling active civil society engagement to challenge inequities [30]. In our case study INGOs challenged government inaction on practice guidelines for delivery of safe-abortions and post-abortion care services, which eventually triggered a transition of practice. A similar example comes from Mexico where globalisation enabled the creation of space for NGOs to work to develop new ways to ensure access to safe abortions for cases qualifying under their quite restrictive laws as well as advocate for the review of punitive laws, building on civic interest and international accords [31].

## Conclusion

We have shown that a number of globalisation forces have contributed to enabling a transition in abortion care in Ghana, from a restrictive interpretation of the law to facilitating more widespread access to legal, safe abortion services through development of policies and guidelines and a slow change in attitudes and practices of health providers. These forces can be categorised as a global governance architecture of reproductive rights-obligations, global communication of ideas and mobility of health providers which facilitate global cultural interaction on the benefits of safe abortion services for reducing consequences of unsafe abortions and tempering negative doctrinal religious beliefs. A global governance architecture that protects the rights of vulnerable people and supports the mitigation of the damaging consequences of morally-contested rights, can contribute a moral weight for the need to act on signed global agreements and create a space for international NGOs to push for accountability on this and demand action. Cross-cultural training opportunities (evident in the highest echelons of the Ghanaian medical hierarchy) also clearly contributed to changing attitudes towards the need for safe abortion services, while the lower cadres had to rely on INGO-initiated training. Thus we have seen that globalisation of information, debate and training experience as well as of international rights frameworks can together create a powerful force for good, even in the face of the global “gag” rule, to protect women and their children from the needless pain and death resulting from unsafe abortions. Now attention should focus on eliminating the use of unsafe services by informing women and girls of their rights to safe, legal abortion care and enhancing demand and good quality services for effective contraception.

## Abbreviations

CAC: Comprehensive Abortion Care
FP: Family planning
GHS: Ghana Health Service
ICPD: International Conference on Population and Development
INGO: International Non-Government Organisation
IPAS: International Pregnancy Advisory Service
MSI: Marie Stopes International
NGO: Non-Government Organisation
PAC: Post abortion care
R3M: Consortium of NGOs working in maternal health
RCH: Reproductive and child health

## Footnotes

1 PRIME I and II are projects carried out in low resource settings like Ghana. PRIME focuses on outputs. Project staffs work with host country ministries of health and other organizations to identify and develop interventions to close performance gaps. The project includes two associate organizations: the American College of Nurse-Midwives (ACNM) and Save the Children. PRIME II was preceded by PRIME I, which was a five-year contract.
